# Preventive effects of Chinese Xinjiang naturally fermented yogurt separated from *Lactobacillus rhamnosus* AFY02 on acute gouty arthritis in mice

**DOI:** 10.1002/fsn3.4268

**Published:** 2024-06-17

**Authors:** Mengwei Wang, Yang Fan, Jing Song, Yanqing Liu, Bihui Liu, Xin Zhao, Wenzhong Wu

**Affiliations:** ^1^ Collaborative Innovation Center for Child Nutrition and Health Development Chongqing University of Education Chongqing China; ^2^ College of Biological and Chemical Engineering Chongqing University of Education Chongqing China; ^3^ Department of Nutrition Chongqing University Jiangjin Hospital Chongqing China; ^4^ Heilongjiang Red Cross Sengong General Hospital Harbin China; ^5^ Harbin Medical University Harbin China

**Keywords:** arthritis, inflammation, *Lactobacillus rhamnosus*, mRNA expression, oxidative stress

## Abstract

*Lactobacillus rhamnosus* AFY02 (LR‐AFY02) is a newly discovered strain isolated and identified from naturally fermented yogurt in Xinjiang, China. This research aims to investigate the mechanism of action of LR‐AFY02 in mice with acute gouty arthritis. We examined the degree of foot swelling, pain threshold, blood biochemical indicators, histopathological changes, and mRNA expression. LR‐AFY02 can decrease the severity of mouse foot edema and raise the pain threshold. LR‐AFY02 can increase the enzyme activity of superoxide dismutase (SOD) and the level of glutathione (GSH) while lowering the enzyme activity of myeloperoxidase (MPO) and the level of malondialdehyde (MDA) in mice with acute arthritis. Interleukin‐6 (IL‐6), IL‐10, interleukin‐1 beta (IL‐1β), and tumor necrosis factor‐alpha (TNF‐α) levels in the blood of mice with acute arthritis are also decreased by LR‐AFY02. Histopathological findings demonstrated that LR‐AFY02 reduced tissue damage in the mouse foot and ankle joints. LR‐AFY02 may suppress the mRNA expression of extracellular signal‐regulated kinase 1/2 (ERK1/2), cyclooxygenase‐2 (COX‐2), prostaglandin E_2_ (PGE_2_), IL‐6, interferon gamma (IFN‐γ), and TNF‐α in the tissues of the ankle joints of mice with acute arthritis. Additionally, LR‐AFY02 has the ability to increase the expression of catalase (CAT), manganese superoxide dismutase (Mn‐SOD), and copper/zinc superoxide dismutase (Cu/Zn‐SOD). As a result, it is clear that *L. rhamnosus* AFY02 is more effective than glucosamine sulfate at preventing acute gouty arthritis.

## Graphical abstract

*Lactobacillus rhamnosus* AFY02 is a newly isolated probiotic from Chinese Xinjiang naturally fermented yogurt. It has a significant intervention effect on mouse acid gouty arthritis and can reduce joint tissue damage.

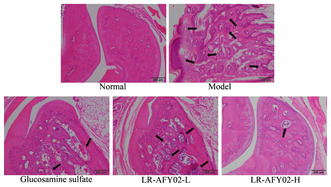

## 1 INTRODUCTION

Gouty arthritis is a recurring inflammatory joint disease caused by disruptions in purine metabolism, resulting in elevated levels of blood uric acid and the subsequent deposition of urate crystals in tissues, including joint capsules, bursae, cartilage, and bone. It manifests through common symptoms such as joint redness, swelling, heat, and pain, making it one of the most prevalent inflammatory joint diseases worldwide. This condition severely impacts patients' sleep quality and daily life due to the experience of joint pain, particularly at night. Disease progression leads to joint stiffness, deformity, and limited mobility (Wilson & Saseen, 2016). Gouty arthritis is associated with high uric acid levels and the formation of uric acid stones. These dynamics can lead to variable degrees of damage to the kidneys and urinary system, resulting in impaired urinary function and potential disease advancement. In certain cases, renal failure may lead to fatalities. As the disease unfolds, gouty arthritis can prompt varying degrees of vascular changes. Consequently, individuals may also present with other conditions, including hypertension or diabetes, in their daily lives. These comorbidities exert impacts on the entire cardiovascular system, heightening the risk of cardiovascular diseases (Zafari et al., 2020).

Located in the northwest region of China, Xinjiang Altay has a unique geographical position as it borders Mongolia and Russia. Due to the long‐standing settlement of various ethnic groups, this region has developed a distinctive environment of ethnic diversity and cultural fusion. Consequently, the dietary habits and culture of the Altay region in China exhibit remarkable diversity. One traditional custom shared by different ethnic groups is the consumption of yogurt, which is widely consumed in the local area. Furthermore, the diverse and complex terrain of Altay, with scorching summers and severe winters, necessitates the movement of livestock to adapt to seasonal climate changes (Liu et al., 2017; Zhang et al., 2016). As a result, local herders have developed a habit of making homemade yogurt and fermented dairy products during the summer to preserve food for the winter. Natural fermented yogurt in the Xinjiang region holds abundant nutritional value and excellent health benefits. Additionally, the fermented microorganisms in yogurt demonstrate strong resistance and diversity, endowing it with broad potential applications and significant value (Zhao et al., 2019). Therefore, further development and utilization of these advantages hold great significance for the growth of the yogurt industry.

*Lactobacillus rhamnosus* GG is a beneficial intestinal bacterium that plays a positive role in the prevention and treatment of gastrointestinal diseases. It has been shown to help prevent and alleviate intestinal infections, antibiotic‐associated diarrhea, enteritis, colorectal cancer, and irritable bowel syndrome (Niu et al., 2023). The supplementation of *L. rhamnosus* GG is beneficial for maintaining a healthy intestinal microbiota and regulating immune responses, including immune reactions in the spleen, intestines, and mesenteric lymph nodes. Moreover, *L. rhamnosus* GG supplementation can reduce the level of intestinal inflammation, thereby preventing the risk of intestinal cancer caused by enteritis (Roller et al., 2004). In addition, a clinical study also showed that *L. rhamnosus* GG has joint softening and swelling effects (Hatakka et al., 2009). *L. rhamnosus* can effectively control monosodium iodoacetate‐induced osteoarthritis in rats, inhibit the severity of pain, and decrease cartilage damage in osteoarthritis. During this process, *L. rhamnosus* also reduces intestinal damage and inflammation, which may be an important way to exert its control over osteoarthritis (Jhun et al., 2021). In this study, the research team collected, homemade naturally fermented yogurt from the Altay region of Xinjiang and isolated and identified *L. rhamnosus* GG from it. Preliminary experiments showed that a strain named *L. rhamnosus* AFY02 (LR‐AFY02) exhibited a survival rate of 144.14% in pH 3.0 simulated gastric fluid (unpublished data), indicating excellent in vitro resistance. Glucosamine sulfate, a drug used for the treatment and prevention of osteoarthritis in various parts of the body, was used as a positive control in this study. The objective of this study is to validate the intervention effect of LR‐AFY02 on gouty arthritis and demonstrate its effectiveness as a probiotic for the prevention and intervention of arthritis.

## 2 MATERIALS AND METHODS

### 2.1 Experimental strain

*L. rhamnosus* AFY02 is a strain of lactic acid bacteria that was isolated and identified from homemade natural fermented yogurt obtained from a herdsman's family in Qiemuerqieke Town, Altay region, Xinjiang, China. The strain was confirmed to be *L. rhamnosus* through 16S rDNA sequencing and was subsequently designated as *L. rhamnosus* AFY02. It has been deposited and preserved at the China General Microbiological Culture Collection Center (CGMCC) under the accession number CGMCC No. 27363.

### 2.2 Animal model

Fifty male BALB/c mice of SPF grade (Shanghai Slaker Laboratory Animal Co. Ltd. Shanghai, China), aged 6 weeks, were randomly assigned to five groups: normal, model, glucosamine sulfate (positive control, Sigma, CA, USA), LR‐AFY02 low concentration experiment (LR‐AFY02‐L), and LR‐AFY02 high concentration experiment (LR‐AFY02‐H), with 10 mice in each group. Mice in the normal and model groups received a daily oral gavage of 0.1 mL/10 g.bw distilled water. Glucosamine sulfate is used as a clinical drug, with a dosage of 1500 mg/d, which is converted into a dosage of 225 mg/kg for mice. The mice in the glucosamine sulfate group were orally gavaged with glucosamine sulfate (Xi'an Quanao Biotechnology Co., Ltd, Xi'an, Shannxi, China) at a dose of 225 mg/kg.bw. According to the recommendations of the World Health Organization (WHO) and the Food and Agriculture Organization of the United Nations (FAO), adults should consume approximately 10 billion probiotics per day, which is equivalent to a dose of approximately 1.5 × 10^9^ CFU/kg.bw for mice, therefore, 1.0 × 10^9^ CFU/kg.bw was chosen as the high dose for this study. The LR‐AFY02‐L and LR‐AFY02‐H groups received oral gavage of LR‐AFY02 experimental strains at doses of 10^8^ CFU/kg.bw and 10^9^ CFU/kg.bw, respectively, for 7 days. Subsequently, all mice were anesthetized with isoflurane, and 50 μL of PBS solution was injected into the right tibiotarsal joint (ankle joint) of mice in the normal group. A suspension was prepared by adding 50 mg of sodium urate (2,6,8‐trihydroxypurine, Sigma) salt to 1 mL of PBS buffer (Zhang et al., 2022). Except for the model group, the right tibiotarsal joint (ankle joint) of the remaining mice was injected with 50 μL of the suspension to induce gouty arthritis. After recovering from anesthesia, all mice continued to receive the corresponding samples orally at the same doses as in the previous 7 days.

### 2.3 Determination of pain threshold by the hot plate method

On the final day of the experiment, the hot plate method (DB026 intelligent hot plate analyzer; Beijing Zhishu Duobao Biotechnology Co. Ltd. Beijing, China) was employed to assess the pain threshold in mice through the application of thermal stimulation. The mice were situated on a hot plate set at a temperature of 55°C, thereby stimulating their paws and evoking a pain response. The time intervals for each mouse to engage in foot licking were meticulously observed and recorded as an indicator of the pain response. This procedure was repeated for five separate groups of mice, with each mouse's duration of right foot licking documented.

### 2.4 Evaluation of joint swelling in mouse

Following the assessment of the mice's pain threshold using the hot plate test, a resting period of 6 h was allowed for all subjects. Subsequently, the cervical dislocation method was employed to euthanize the mice. Then, a digital caliper was utilized to measure the diameter of the right ankle, enabling an evaluation of the extent of ankle swelling.

### 2.5 Measurement of oxidative state in mouse ankle joint tissues

In order to assess the oxidative status of the mouse ankle joint tissues, 0.1 g of tissue from the right ankle joint was weighed. Subsequently, 0.9 mL of physiological saline was added to the tissue. To separate the supernatant from the mixture, it was centrifuged at 4000 rpm for 10 min (Long et al., 2022). The levels of important markers, including MPO, SOD, GSH, and MDA, in the supernatant of the tissue homogenate were measured using specific assay kits (Nanjing Jiancheng Science and Technology Co, Ltd, Nanjing, Jiangsu, China).

### 2.6 Measurement of inflammatory cytokines in mouse serum

Mouse whole blood was obtained through retro‐orbital bleeding. The collected samples were centrifuged at 4000 rpm for 10 min at 4°C to separate the serum (Long et al., 2022). The levels of IL‐6, IL‐10, IL‐1β, and TNF‐α inflammatory markers in the serum were measured using ELISA assay kits (Thermo Fisher, MA, USA).

### 2.7 Histopathological examination of mouse tissues

The ankle joint tissue was excised from mice and fixed in a 10% paraformaldehyde solution. Subsequently, the tissue was decalcified for a period of 20 days using a decalcification solution consisting of a mixture of concentrated hydrochloric acid, formalin, and distilled water in a ratio of 10:9:81. After decalcification, the ankle joint tissue samples were embedded in paraffin, sectioned, and stained with hematoxylin and eosin (H&E, Beyotime Biotechnology, Shanghai, China). The stained sections were then examined using an optical microscope (BX53, Olympus, Tokyo, Japan) to analyze any pathological changes in the tissue (Hu, Chen, et al., 2022).

### 2.8 Measurement of mRNA expression in mouse ankle joint tissue

To determine mRNA expression in ankle joint tissue, 0.1 g of tissue was weighed and placed in a container. 0.9 mL of physiological saline was added and thoroughly mixed. Subsequently, 0.1 mL of RNAzol solution (Thermo Fisher) was added to extract RNA from the mouse ankle joint tissue. The absorbance values at 260 nm and 280 nm were measured using a microplate spectrophotometer (Nano‐100, Allsheng, Hangzhou, Zhejiang, China) to determine the purity and concentration of RNA, which was then adjusted to 1 μg/μL. Reverse transcription was performed to generate cDNA, and a reaction system was prepared containing 1 μL of cDNA. The reaction mixture included 10 μL of SYBR Green PCR Master Mix, 7 μL of sterile distilled water, and 1 μL each of forward and reverse primers (Thermo Fisher). The reaction was performed using a quantitative PCR instrument (Stepone Plus, Thermo Fisher) under the following conditions: 60 s at 95°C, 15 s at 95°C for 40 cycles, 30 s at 55°C, and 35 s at 72°C. Finally, the target gene was analyzed using the 2^−ΔΔCt^ method with glyceraldehyde‐3‐phosphate dehydrogenase (GAPDH) as the internal reference (Hu, Luo, et al., 2022; Zhou et al., 2021).

### 2.9 Data analysis

The experiments were conducted in triplicate, and the results were calculated by determining the average value and standard deviation. The mean ± standard deviation was used to present the experimental results. Furthermore, a one‐way analysis of variance (ANOVA) was conducted to assess if there were significant differences between the groups (*p* < .05).

## 3 RESULTS

### 3.1 Degree of ankle joint swelling in mice

According to Table 1, the model group demonstrated the highest degree of ankle joint swelling. It was observed that both glucosamine sulfate and the LR‐AFY02 strain significantly reduced (*p* < .05) the degree of mild ankle joint swelling caused by arthritis. Additionally, the LR‐AFY02 strain at a high concentration (LR‐AFY02‐H) exhibited the most effective results.

**TABLE 1:** Degree of ankle joint edema in each group of mice.

Group	Normal	Model	Glucosamine sulfate	LR‐AFY02‐L	LR‐AFY02‐H
Degree of ankle joint edema (mm)	2.39 ± 0.11^b^	3.85 ± 0.35^a^	3.17 ± 0.26^ab^	3.29 ± 0.19^ab^	2.93 ± 0.21^b^

*Note*: ^a, b^Lower case letters that are the same indicate no significant difference between the corresponding two groups, while different lower case letters indicate a significant difference between the corresponding two groups (*p* < .05).

### 3.2 Mouse pain threshold

According to the findings presented in Table 2, it can be observed that the pain threshold (time) of the normal group, of mice was the highest, in contrast to the model group where it was the shortest. The administration of both glucosamine sulfate and the LR‐AFY02 strain led to a significant (*p* < .05) increase in pain threshold when compared to the model group. Furthermore, the high concentration of the LR‐AFY02 strain (LR‐AFY02‐H) exhibited a more pronounced effect on enhancing the pain threshold compared to glucosamine sulfate and the low concentration of the LR‐AFY02 strain (LR‐AFY02‐L).

**TABLE 2:** Pain threshold of each group of mice.

Group	Normal	Model	Glucosamine sulfate	LR‐AFY02‐L	LR‐AFY02‐H
Licking foot time on a hot plate (s)	22.1 ± 1.7^a^	6.7 ± 2.5^c^	12.5 ± 3.3^ab^	11.3 ± 2.4^ab^	17.7 ± 3.1^b^

*Note*: ^a, b^Lower case letters that are the same indicate no significant difference between the corresponding two groups, while different lower case letters indicate a significant difference between the corresponding two groups (*p* < .05).

### 3.3 MPO activity, SOD enzyme activity, and levels of GSH and MDA in the ankle joint tissue of mice

Based on the data presented in Table 3, it is evident that the normal group of mice exhibited the lowest MPO enzyme activity and MDA level in the ankle joint tissue, while displaying the highest SOD enzyme activity and GSH level. Conversely, the model group of mice demonstrated an inverse pattern, with the ankle joint tissue showing the highest MPO enzyme activity and MDA level, while displaying the lowest SOD enzyme activity and GSH level. Upon administering glucosamine sulfate and the LR‐AFY02 strain via oral gavage, a notable decrease in MPO enzyme activity and MDA level, along with an increase in SOD enzyme activity and GSH level, was observed in the ankle joint tissue of mice with arthritis. Notably, the LR‐AFY02 strain at a high concentration (LR‐AFY02‐H) exerted the most pronounced effect.

**TABLE 3:** The MPO and SOD enzyme activity and GSH and MDA levels in mouse ankle joint tissue.

Group	MPO (U/g)	SOD (U/mg)	GSH (mg/g)	MDA (nmol/mg)
Normal	0.36 ± 0.02^d^	6.13 ± 0.09^a^	46.11 ± 2.86^a^	5.35 ± 0.55^d^
Model	2.46 ± 0.17^a^	1.14 ± 0.28^d^	17.21 ± 2.27^d^	26.31 ± 1.22^a^
Glucosamine sulfate	1.79 ± 0.21^b^	2.61 ± 0.37^c^	30.57 ± 1.82^c^	20.03 ± 1.31^b^
LR‐AFY02‐L	1.92 ± 0.21^b^	2.42 ± 0.39^c^	29.42 ± 3.02 ^c^	20.59 ± 1.73^b^
LR‐AFY02‐H	0.74 ± 0.06^c^	4.82 ± 0.28^b^	38.81 ± 2.51^b^	12.53 ± 1.44^c^

*Note*: ^a–d^Lower case letters that are the same indicate no significant difference between the corresponding two groups, while different lower case letters indicate a significant difference between the corresponding two groups (*p* < .05).

### 3.4 Levels of IL‐6, IL‐10, IL‐1β, and TNF‐α cytokines in mouse serum

According to Table 4, it is evident that the levels of IL‐6, IL‐1β, and TNF‐α cytokines were lowest in the serum of the normal group of mice. Conversely, the induction of arthritis in mice (model group) resulted in a significant increase in the levels of IL‐6, IL‐1β, and TNF‐α cytokines in the ankle joint tissue (*p* < .05). Both the glucosamine sulfate and LR‐AFY02 strains effectively suppressed the elevation of IL‐6, IL‐1β, and TNF‐α cytokine levels, with the LR‐AFY02 strain at a high concentration (LR‐AFY02‐H) exhibiting the strongest inhibitory effect. Moreover, the normal group of mice demonstrated the highest level of the IL‐10 cytokine, whereas the model group had the lowest level. Compared to the model group, both the LR‐AFY02 strain and glucosamine sulfate exhibited the ability to increase IL‐10 levels in mouse serum, with the LR‐AFY02‐H strain demonstrating the most substantial effect.

**TABLE 4:** The levels of IL‐6, IL‐10, IL‐1β, and TNF‐α cytokines in mouse serum.

Group	IL‐6 (ng/mL)	IL‐10 (ng/mL)	IL‐1β (ng/mL)	TNF‐α (ng/mL)
Normal	52.55 ± 0.79^d^	35.35 ± 1.56^a^	12.29 ± 0.82^c^	116.30 ± 5.10^d^
Model	88.54 ± 8.64^a^	24.16 ± 1.88^d^	24.48 ± 1.69^a^	246.33 ± 20.79^a^
Glucosamine sulfate	65.26 ± 4.48^b^	27.91 ± 2.09^c^	20.72 ± 1.30^ab^	201.74 ± 17.69^b^
LR‐AFY02‐L	70.38 ± 6.43^b^	26.93 ± 2.09^c^	21.50 ± 2.08^ab^	208.48 ± 7.15^b^
LR‐AFY02‐H	58.10 ± 3.09^c^	30.78 ± 2.64^b^	15.11 ± 1.38^b^	147.42 ± 9.83^c^

*Note*: ^a–d^Lower case letters that are the same indicate no significant difference between the corresponding two groups, while different lower case letters indicate a significant difference between the corresponding two groups (*p* < .05).

### 3.5 Histopathological observations of mouse ankle joint tissue

**FIGURE 1:**
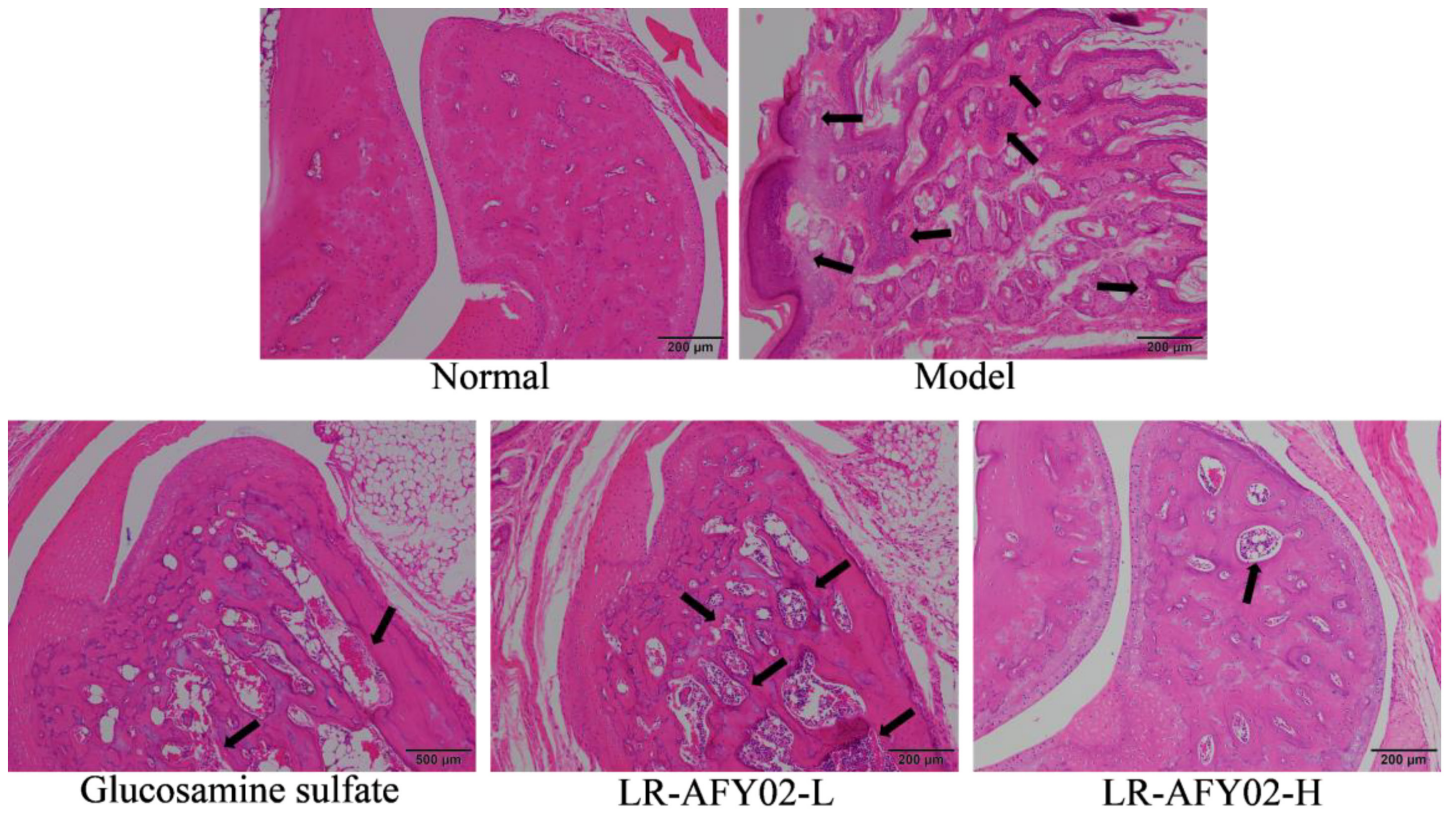
The pathological observation of the mouse ankle joint (×100).

Based on the findings in Figure 1, it is evident that the ankle joint sections of the model group show a substantial increase in the infiltration of inflammatory cells. Nonetheless, the administration of glucosamine sulfate and the LR‐AFY02 strain effectively regulates the levels of inflammation in arthritic mice, resulting in a reduction in the infiltration of inflammatory cells. Moreover, as the dosage of the LR‐AFY02 strain increases, the inhibitory effect on ankle joint inflammation improves, with the LR‐AFY02‐H strain demonstrating superior efficacy compared to glucosamine sulfate.

### 3.6 mRNA expression in the ankle joint tissue of mice

**FIGURE 2:**
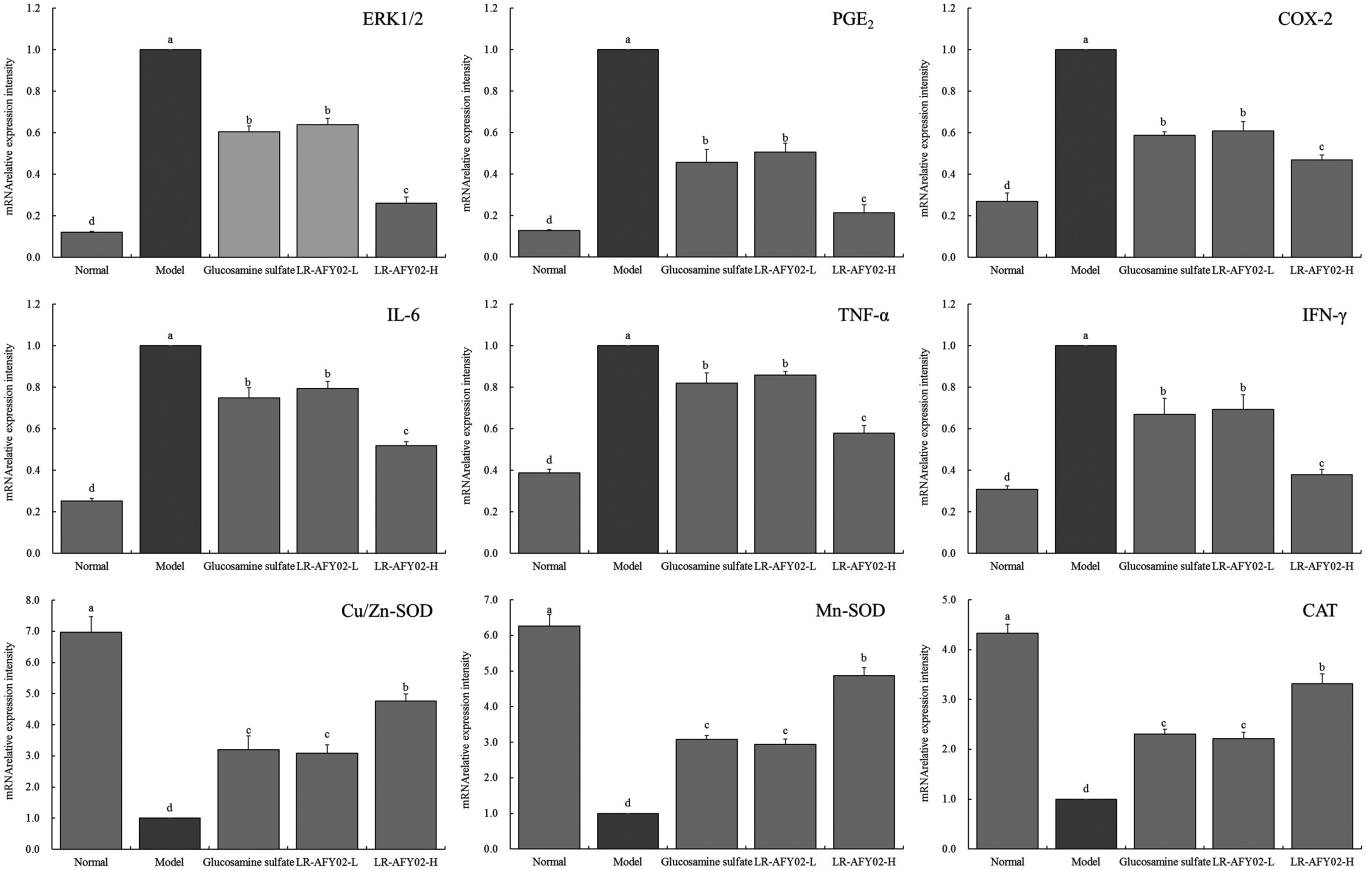
mRNA expression in mouse ankle joint tissue. ^a–d^Lower case letters that are the same indicate no significant difference between the corresponding two groups, while different lower case letters indicate a significant difference between the corresponding two groups (*p* < .05).

Based on the findings presented in Figure 2, it is evident that the mRNA expression levels of ERK1/2, COX‐2, PGE2, IL‐6, IFN‐γ, and TNF‐α are highest in the ankle joint tissue of mice in the model group, while they are weakest in the normal group. The administration of glucosamine sulfate and the LR‐AFY02 strain effectively downregulate the expression of ERK1/2, COX‐2, PGE_2_, IL‐6, IFN‐γ, and TNF‐α in the ankle joint tissue of arthritic mice. Notably, the LR‐AFY02 strain demonstrates the most potent ability to downregulate the expression of ERK1/2, COX‐2, and PGE_2_. Conversely, the ankle joint tissue of mice in the normal group exhibits the highest mRNA expression levels of Cu/Zn‐SOD, Mn‐SOD, and CAT, whereas these expressions are considerably lower in the model group. In comparison to the model group, the administration of the LR‐AFY02 strain leads to an upregulation in the expression of Cu/Zn‐SOD, Mn‐SOD, and CAT, with the LR‐AFY02‐H group demonstrating a more pronounced expression than both the LR‐AFY02‐L group and the glucosamine sulfate group.

## 4 DISCUSSION

Gouty arthritis is characterized by the sudden and severe onset of joint pain, which results in inflammation in the local joint capsule, bursa, cartilage, bone, and other surrounding tissues. The activation of inflammation and the generation of various oxygen‐free radicals, along with oxidative stress, are closely associated with joint damage during acute flare‐ups of gouty arthritis. Immune and inflammatory processes are key contributors to the elevated levels of oxygen‐free radicals. Consequently, regulating the body's inflammatory response and oxidative stress processes can play a critical role in managing arthritis (Umar et al., 2012). The gut microbiota and its metabolites are of utmost importance in the host's immune system. They have a close relationship with inflammation and imbalanced oxidative stress within the body, and are intimately linked to the occurrence and development of arthritis (Liu & Yu, 2019). Clinical studies have revealed significant differences in the quantity and composition of the gut microbiota between patients with arthritis and healthy individuals. Dysbiosis of the gut microbiota can contribute to the onset and progression of arthritis, while the ongoing progression of arthritis can further worsen the dysbiosis, leading to a reciprocal interaction between the two factors (Fang & Liu, 2017). Research has demonstrated that the gut microbiota can influence the host's immune functions through various mechanisms, including regulating immune cell differentiation, utilizing molecular mimicry mechanisms, and inducing the production of inflammatory mediators. These effects have the potential to trigger the development of autoimmune diseases. Certain approaches, such as probiotics and fecal microbiota transplantation, have shown promise in restoring a healthy gut microbiota, improving dysbiosis, and potentially preventing the onset and progression of arthritis, as well as ameliorating its associated symptoms (Aggarwal et al., 2017).

During the acute phase of arthritis, characteristic symptoms such as joint swelling, pain, and restricted mobility are observed. To evaluate the severity of arthritis, various studies have utilized methods that involve observing foot swelling and measuring pain thresholds in experimental animals (Teranaka et al., 2014). Therefore, in this study, an assessment of foot swelling in mice was conducted using visual observation, and the pain threshold was measured using a hot plate analgesia meter. The experimental results further confirmed that *L. rhamnosus* AFY02 effectively prevents foot swelling and reduces the decrease in pain threshold caused by acute arthritis in mice.

Myeloperoxidase (MPO) possesses oxidative properties that can trigger reactive oxygen species (ROS) generation and oxidative stress reactions. ROS can cause cellular and tissue oxidative damage, exacerbating inflammation and injury in joint tissues. Furthermore, MPO can activate inflammatory cells and induce the production of inflammatory mediators, including cytokines and chemotactic factors, thereby intensifying the inflammatory response in arthritis. In arthritis, inflammation leads to oxidative stress, resulting in an excessive generation of intracellular ROS that surpasses the cell's antioxidant capacity. These ROS can damage joint tissues and contribute to the inflammation and pain associated with arthritis (Edwards et al., 1988).

SOD and GSH play a crucial role in scavenging ROS, reducing oxidative stress, and protecting joint tissues from ROS‐induced damage (Wetscher et al., 1995). In arthritis, both inflammation and oxidative stress can elevate lipid peroxidation, leading to increased MDA production. Excessive accumulation of MDA can negatively impact joint tissues, exacerbating symptoms and inflammation in arthritis. Moreover, MDA can activate the generation of inflammatory cells and mediators, promoting the degradation of bone, cartilage, and joint tissues (Yuan et al., 2014). The experimental results demonstrate that the ability of *L. rhamnosus* AFY02 to mitigate oxidative stress may be a crucial approach for preventing acute arthritis.

Inflammatory cytokines play a significant role in tissue damage within the body. Pathological changes trigger tissue oxidation and inflammation, resulting in the release of various inflammatory mediators during oxidative stress. Abnormal increases in pro‐inflammatory cytokines, including IL‐6, IL‐10, IL‐1β, and TNF‐α, can exacerbate the progression of tissue damage (Tripathy et al., 2021). IL‐6 is a cytokine that plays a crucial role in the inflammatory process. It stimulates synovial cells and other inflammatory cells to produce inflammatory mediators like IL‐1β and TNF‐α, thus intensifying inflammation in the joints. Furthermore, IL‐6 promotes the proliferation of synovial cells and angiogenesis, further aggravating inflammation and tissue destruction. IL‐6 also regulates immune cell differentiation and function, impacting T cells, B cells, and macrophages. Excessive production of IL‐6 in arthritis can activate and proliferate inflammatory T cells and B cells, triggering immune reactions and autoantibody production, thereby worsening arthritis (Gottenberg et al., 2012). IL‐10 is an anti‐inflammatory cytokine primarily responsible for inhibiting inflammation and maintaining immune system balance. IL‐10 suppresses the production of inflammatory mediators, including IL‐1β and TNF‐α, reducing the intensity of inflammatory reactions and alleviating symptoms and inflammation in arthritis. Conversely, a deficiency in IL‐10 can lead to excessive immune cell activation and sustained inflammatory responses, exacerbating arthritis progression (Tripathy et al., 2021). IL‐1β plays a critical role in the development of arthritis as it initiates an inflammatory response and promotes the activation and proliferation of inflammatory cells in joints. IL‐1β stimulates synovial and cartilage cells to release other inflammatory cytokines, such as TNF‐α and IL‐6, further intensifying inflammation in the joints. These inflammatory factors contribute to increased joint inflammation, resulting in swelling, pain, and functional impairment. Additionally, IL‐1β promotes osteoporosis and cartilage damage in joints, leading to further joint degradation (Czerny et al., 2010). TNF‐α induces inflammation, attracting inflammatory cells to the joints and promoting their activation and proliferation. It stimulates synovial and cartilage cells to produce other inflammatory mediators like IL‐1β, IL‐6, and IL‐8, exacerbating the inflammatory response within the joints and causing joint swelling, pain, and functional impairment (Redlich et al., 2002). The experimental results of this study also demonstrate that *L. rhamnosus* AFY02 has the ability to regulate inflammatory cytokines, thus exerting a preventive effect on acute arthritis.

The ERK1/2 signaling pathway and the COX‐2/PGE_2_ pathway interact with each other. Upon activation, ERK1/2 can directly or indirectly regulate the expression and activity of COX‐2, thereby impacting PGE2 synthesis. ERK1/2 is an extensively studied cell signaling molecule that participates in various biological processes such as cell proliferation, differentiation, and inflammation. In arthritis, the activation levels of ERK1/2 are often significantly increased, which may be associated with the proliferation of inflammatory cells, synthesis of inflammatory mediators, and damage to joint tissues (Marotte et al., 2010). COX‐2 is a crucial enzyme involved in the synthesis of prostaglandins, including PGE2, in the inflammatory response. In arthritis, there is often an upregulation in COX‐2 expression, leading to increased PGE_2_ synthesis (Kumar et al., 2015). PGE_2_ plays a pro‐inflammatory role in arthritis and contributes to pathological processes such as arthritis pain, activation of inflammatory cells, and skeletal destruction (Bouffi et al., 2010). IFN‐γ can stimulate inflammatory cells to produce inflammatory mediators responsible for arthritis symptoms, such as TNF‐α, IL‐1, and various chemokines. These inflammatory mediators can trigger the inflammatory response and tissue damage in arthritis. IFN‐γ plays a significant role in the inflammatory response by regulating the function of inflammatory cells and promoting the production of inflammatory mediators, thereby influencing the development of arthritis (Justa et al., 2014). Cu/Zn‐SOD and Mn‐SOD are the main antioxidant enzymes involved in cellular clearance of superoxide radicals, thereby maintaining cellular redox homeostasis. Cu/Zn‐SOD is primarily located in the cytoplasm, while Mn‐SOD is predominantly found in the mitochondria. Oxidative stress induced by inflammation is an important mechanism in arthritis development. Increased activity of Cu/Zn‐SOD and Mn‐SOD can inhibit the accumulation of intracellular superoxide radicals and alleviate oxidative stress and inflammatory responses, thus serving as a preventive and intervention strategy in arthritis (Kurz et al., 2002). CAT is predominantly present in the cytoplasm and functions as a vital enzyme in clearing hydrogen peroxide within cells. Hydrogen peroxide is also one of the major products of oxidative stress induced by arthritis, and at high concentrations, it can cause lipid peroxidation and protein oxidation damage in joints (Sen et al., 2009). *L. rhamnosus* AFY02 effectively regulates joint tissue mRNA, shifting the expression of arthritis‐related genes toward a favorable trend for the body's recovery. This highlights its role in the regulatory effect on acute arthritis.

Currently, there are only a few studies on the effect of *L. rhamnosus* GG on arthritis, among which *Lacticeibacillus rhamnosus* GG, as one of the most extensively studied strains, also has an effect on arthritis. In this study, *L. rhamnosus* AFY02 was a newly discovered strain, and its effect on arthritis was observed for the first time. At the same time, its mechanism was preliminarily elucidated by observing its regulation of inflammation and oxidative stress, providing a new probiotic for the application of *L. rhamnosus* in arthritis.

## 5 CONCLUSIONS

In this study, ankle arthritis was successfully induced in all groups of mice except for the control group following sodium urate salt induction. *L. rhamnosus* AFY02 was found to effectively reduce ankle swelling and alleviate pain in the mice. Observations of serum and tissue samples revealed that LR‐AFY02 had the ability to inhibit inflammation and oxidative damage in the ankle joints. Further mRNA expression experiments demonstrated that LR‐AFY02 could suppress ankle joint swelling and pain at the molecular level, resulting from arthritis. These findings indicate that *L. rhamnosus* AFY02 is a beneficial probiotic with promising intervention effects on gouty arthritis. The experimental results provide valuable insights for the development of high‐quality probiotics for arthritis regulation and contribute to the creation of probiotic products with autonomous intellectual property rights. However, these results still require validation through further clinical studies, and deeper exploration of *L. rhamnosus* AFY02 is warranted in the future.

## AUTHOR CONTRIBUTIONS

**Mengwei Wang:** Writing – original draft (equal). **Yang Fan:** Writing – original draft (equal). **Jing Song:** Data curation (equal). **Yanqing Liu:** Data curation (equal). **Bihui Liu:** Formal analysis (equal). **Xin Zhao:** Writing – review and editing (equal). **Wenzhong Wu:** Writing – review and editing (equal).

## FUNDING INFORMATION

This research was supported by the General Program of the Natural Science Foundation of the Chongqing (CSTB2023NSCQ‐MSX0882), the Science and Technology Project of Chongqing Education Commission (KJZD‐M202201601), and the Chongqing Returned Overseas Chinese Entrepreneurship and Innovation Support Program Project (cx2022030).

## CONFLICT OF INTEREST STATEMENT

The authors declare no conflict of interest relevant to this article.

## ETHICS STATEMENT

The animal experiment was approved by the Animal Experiment Ethics Committee of the Collaborative Innovation Center for Child Nutrition and Health Development, Chongqing University of Education (Chongqing, China), with the approval number 2022070035B.

## Data Availability

The data are available upon request from the authors.
